# People Believe and Behave as if Consumers of Natural Foods Are Especially Virtuous

**DOI:** 10.3389/fpsyg.2018.01823

**Published:** 2018-09-27

**Authors:** Zoe Taylor, Richard J. Stevenson

**Affiliations:** Department of Psychology, Macquarie University, Sydney, NSW, Australia

**Keywords:** natural foods, morality, trust game, open response, behavioral test, virtue

## Abstract

We examined here whether people believe consumers of natural foods are more virtuous than consumers of unnatural foods. In Study 1, we asked student participants (*n* = 84; 77 female, *M* age = 19.5) to form an impression of another person based solely upon whether they ate natural or unnatural foods, these being determined in a pilot survey. On an open-response format, participants reported more positive moral and health traits in consumers of natural foods. These findings were further confirmed using rating-based evaluations. In Study 2, we determined if this belief in the virtuousness of natural food consumers translated into behavior. Student participants (*n* = 40; 25 female, *M* age = 20.2) played a trust game, exchanging tokens with a fictitious player. Incidental diet information about the fictitious player was provided, with participants in one group playing against a natural food consumer and those in another against an unnatural food consumer. Participants who played against a natural food consumer behaved as if they trusted this person more, and their performance on the game was predicted by how moral they felt the fictitious player was, but not by other attributes such as health. These findings suggest that people believe consumers of natural food are more virtuous, and we suggest this is driven by the altruistic attitudes that people believe to be associated with natural food consumption.

## Introduction

What people eat has moral overtones (Stein and Nemeroff, 1995). This can be seen in religious belief systems, where some seek to demarcate the pure from the profane (e.g., kashrut, halal), and others act to minimize animal suffering (e.g., Hinduism, Buddhism). The latter is also evident in secular societies – vegetarianism, veganism (e.g., Rozin et al., 1997) – although its motivation can also reflect the pursuit of physical and mental health. This health goal, which typically encompasses a broader set of lifestyle factors including diet, exercise, and spiritual type practices (e.g., yoga, etc.) – but devoid of links to organized religion – is also closely linked to morality. Here, the pursuit of health suggests both the mental discipline of the practitioner and the purifying effect of their practices, and its converse, ill health, suggests more negative moral connotations (e.g., Conrad, 1994; Hoverd and Sibley, 2007). Located in the midst of consumer concerns over animal welfare and the pursuit of health and wellbeing, is the growing preference for natural foods (Rozin et al., 2004, 2012). As we outline below, consuming a diet composed of natural foods – as with a vegetarian, healthy or religiously orientated diet – might in the eyes of others imbue its consumer with virtue (i.e., positive moral overtones). The aim of the two studies reported in this manuscript was to test this idea using both novel open-response format (Study 1) and behavioral (Study 2) approaches.

There is no formal definition (legal or otherwise) of what constitutes a natural food. As such, any definition is based upon the sort of properties that people perceive these foods to have. This may include one or more of the following: (1) a resemblance to organic foods; (2) relating positively to nature (biophillia; Wilson, 1984); (3) an absence of mechanical processing and additive chemicals (preservatives, colors, agrochemicals; e.g., Siegrist and Sutterlin, 2017); (4) being eco- and animal friendly; and (5) being fair trade (e.g., Rozin, 2005, 2006; Kniazeva, 2006; Hasselbach and Roosen, 2015). Extensive market research, using various measures of naturalness in many different cultures, suggests that consumers prefer natural foods to ones that do not possess this quality (i.e., processed food, additives, products of factory farming, etc.; Nielsen Global Health and Welfare Survey, 2015; Nielsen Global Health and Ingredient-Sentiment Survey, 2016). The source of this preference for naturalness may have both instrumental and ideational components (Rozin et al., 2004; Ricci et al., 2018). Many consumers claim that natural foods are healthier and tastier. However, there is little support for the idea of sensory or health differences when comparable foods are contrasted (e.g., organically grown vegetables vs. commercially farmed vegetables; e.g., Bourn and Prescott, 2002; Hemmerling et al., 2016). Nonetheless, as natural foods tend to encompass foods that are generally acknowledged to be healthier to consume (i.e., fruits, vegetables, whole grains), a natural diet is likely to be more consistent with current dietary guidelines (Siipi, 2013). That aside, ideational components are likely to be more dominant in driving a preference for natural foods, most notably in the perception that they are inherently better than foods not deemed as natural (Rozin et al., 2004).

There are at least three reasons to think that natural foods – and hence their consumers – should be linked with more positive moral attributes. First, two studies indicate an association between eating natural foods and morality. Pohjanheimo et al. (2010) observed that greater consumption of natural foods was positively associated with greater reported desire to protect the welfare of people, animals, and nature. Makiniemi et al. (2011) used a word association task to explore the concepts linked to “ethical” and “unethical” food. Naturalness emerged as a core component of what constitutes an ethical food. In sum, and noting the limited literature, both studies suggest that positive moral overtones are linked to natural foods.

A further reason to think that natural food consumption should be associated with virtue concerns the beliefs, which may drive a preference for them. Some of these beliefs may relate to modern agricultural practices (e.g., Lockie et al., 2002). Modern farming is often perceived as ecologically harmful and cruel, especially as it pertains to meat, dairy, and egg production. Culturally, these concerns are reflected in the emerging ethical eating movement (e.g., Johnston et al., 2011) and in films such as *Food Inc* and *Ingredients*. Similar concerns have been identified as motivational drivers for people adopting a vegetarian diet (Rozin et al., 1997). Indeed, non-vegetarians are also well aware of such moral motives for vegetarianism (Ruby and Heine, 2011). The key issue here is people’s concerns – which are in part *moral concerns* – over industrialized agriculture, with this serving as a motive for preferring foods generated by more *natural* processes.

The third reason for thinking there is a connection between natural food and morality comes from the link between natural foods and health foods, and the broader pursuit of health and wellbeing as a *moral* undertaking. A significant motive for consuming natural foods is their purported health benefits (Roininen et al., 2001; Rozin et al., 2004). Several studies have demonstrated that consumers of health foods (e.g., low fat, vegetables, and whole grains), are perceived more positively by others than consumers of less healthy, processed, and high-fat foods. These positive attributes include being judged to be healthier, intelligent, feminine, educated, and of especial interest here, being a more virtuous person (Fries and Croyle, 1993; Mooney et al., 1994; Stein and Nemeroff, 1995; Barker et al., 1999; Oakes and Slotterback, 2004/2005). More generally, similar associations between good health, pursuit of health-related behaviors, and positive moral traits, have been identified in several other studies (e.g., Conrad, 1994; Hoverd and Sibley, 2007). In sum, natural foods are considered healthy foods, and healthy food consumption – and health-orientated behaviors in general – are deemed virtuous.

Based upon these findings, we suggest that people will judge consumers of natural food as being more virtuous than people who consume unnatural foods – that is processed, junk and fatty foods, etc. In addition, we would also expect that consumers of natural food to be judged as possessing the type of attributes reported for consumers of health foods, namely being healthier, as well as having certain demographic associations (e.g., feminine, educated, wealthy, and older; Fries and Croyle, 1993; Mooney et al., 1994; Stein and Nemeroff, 1995; Barker et al., 1999; Oakes and Slotterback, 2004/2005). These predictions were explored in Study 1 using an open-response format to avoid cueing bias (i.e., asking about morality may simply serve to bring this to mind) followed by a more typical closed-response format. A further prediction was examined in Study 2. If people believe that consumers of natural food are more virtuous than consumers of unnatural food, then presumably they should trust the former more than the latter. To test this hypothesis, we undertook an experimental study, using the Trust Game (Hillebrandt et al., 2011), and manipulated participants beliefs about the other (fictitious) player by providing incidental information about their diet – namely whether they consumed natural or unnatural foods.

## Study 1

Study 1 examined whether natural food consumers are judged to possess more positive moral traits than consumers of unnatural foods. In addition, it also explored whether consumption of natural foods is associated with positive health-related constructs (e.g., active, fit, healthy) and demographic characteristics (e.g., feminine, older, educated, wealthy; Fries and Croyle, 1993; Mooney et al., 1994; Stein and Nemeroff, 1995; Barker et al., 1999; Oakes and Slotterback, 2004/2005). The basic approach involved presenting participants with pictorial sets of foods. These were composed of natural sets, unnatural sets and mixed sets (i.e., both natural and unnatural foods), the last mentioned being included to make the purpose of the study less apparent. Based on these items, participants were asked to evaluate the likely characteristics of people who eat them. Two evaluation approaches were adopted. The first – and always presented first – used an open-ended format, to see if participants spontaneously identified moral characteristics in natural food consumers in the absence of any cues (i.e., rating labels). The second more traditional approach used rating scales. Twenty-five rating scales were drawn from Stein and Nemeroff (1995) and Hellyer et al. (2014), to reflect the two categories described above, the demographic items, and distractor items. The Moral category was composed of eight ratings, with seven drawn from Stein and Nemeroff (1995) morality and related scales (tolerant, monogamous, considerate, virtuous, pure, disciplined, follows norms), and one from Hellyer et al. (2014) study (high aspirations), the aim being to reflect a range of attributes that would capture participant’s views of moral-related behavior. The Health category was composed of five ratings (health conscious, healthy, active, thin, and attractive). Four demographic related variables were collected (gender, age, education, and wealth) and eight distractor ratings (intelligent, likeable, neophobic, plans ahead, practical, hard-working, methodical, and talkative). Finally, before undertaking Study 1, we conducted a pilot to select food types for use here and in Study 2. This was to ensure that the selected foods reflected Australian participants’ views on what was and was not a natural food (or beverage).

### Methods

#### Participants

Participants were Macquarie University students who took part for course credit. Desired sample size was conservatively based upon powering the study to detect a small to medium effect size (*d* = 0.35, with power at 80%, requires a sample size of 79 on 2 df), as we had no prior basis on which to estimate the frequency with which participants would spontaneously identify moral themes in the open-ended response format. Eighty-four individuals (77 women) completed the survey. They were aged between 17 and 49 years (*M* = 19.5, *SD* = 4.7). The majority described their diet as omnivorous (84.5%), with a minority of vegetarians (8.3%), vegans (4.8%), and “others” (2.4%). This study was carried out in accordance with the recommendations of the Macquarie University Human Research Ethics Committee (MQ HREC). The protocol was approved by the MQ HREC. All subjects gave written informed consent in accordance with the Declaration of Helsinki.

#### Stimuli

An important consideration was to identify foods that participants would consider as natural (and unnatural). To this end we conducted a pilot to identify such foods. Twenty-one individuals (61.9% females) participated (*M* age = 21.8, *SD* = 2.3) in a 30-min online survey. A list of 106 foods and drinks were compiled, which we felt were high, intermediate, or low in naturalness, drawn from item categories taken from the online website of a leading supermarket: “Fruit and Vegetables,” “Drinks,” “Bread and Bakery,” “Dairy and Eggs,” “Meat,” “Confectionary and Snacks,” “Breakfast Items,” “Staple Pantry Items,” “Pre-Prepared Meals” and “Canned Food.” Participants were shown a description and image of a food/drink, with this being followed by three ratings (using 7-point category scales) Naturalness (anchors [1] “Extremely Natural” to [7] “Extremely Unnatural”), Healthiness (anchors [1] “Extremely Healthy” to [7] “Extremely Unhealthy”), and Level of Processing (anchors [1] “Processed in a Factory” to [7] “Not Processed in a Factory”). The remaining food/drink items were presented and rated in the same manner, with a different random presentation order used for each pilot participant.

Naturalness, healthiness, and level of processing were all positively correlated. Average naturalness ratings for each item were computed, and these were then ranked from those judged the most natural (a lime, *M* = 1.1/7) to the most unnatural (Cheezels, *M* = 6.7/7). Based on these rankings, three categories were formed – Natural, Unnatural, and Mixed Food Sets – consisting of both natural and unnatural food items. Within each category, food items were further sorted into sets of six based on the criteria that each set was approximately balanced on freshness (e.g., vegetables/dried goods), type of processing (e.g., none/canned) and food type (e.g., sweet/savory). This was achieved by categorizing each food item based on these qualities and randomly allocating them across sets. The resulting food sets are presented in **Table 1**.

**Table 1:** The food stimuli used in Study 1.

Set 1 Natural	Set 2 Natural	Set 3 Natural	Set 4 Natural
**Natural food sets**			
Lime	Raspberries	Pineapple	Banana
Broccoli	Sugar snap peas	Frozen spinach	Avocado
Free range eggs	Full cream milk	Greek yoghurt	Fresh caged eggs
Raw mixed nuts	Whole chicken	Lamb leg	Chicken breast
Green tea	Orange juice	Coconut water	Pressed juice
Porridge oats	Honey	Almond spread	Coconut oil

**Set 1 Mixed**	**Set 2 Mixed**	**Set 3 Mixed**	**Set 4 Mixed**

**Mixed food sets**			
Pink lady apples	Dried apricots	Bacon	Beef mince
Beetroot	Spinach bunch	Frozen baby peas	Canned corn
Pure cream	Feta	Vegetable oil	Ciabatta loaf
Peach ice tea	Ginger beer	Dairy milk	Mud cake
Mi goreng	Frozen pizza	Sweet potato chips	LCM bar
Kettle potato chips	Coco pops	Cocktail frankfurts	French fries

**Set 1 Unnatural**	**Set 2 Unnatural**	**Set 3 Unnatural**	**Set 4 Unnatural**

**Unnatural food sets**			
Coke	Powerade	Red Bull	Coke Zero
Tuna mornay	Old El Paso meal	Latina lasagne	Hamburger
CC’s corn chips	Shapes	Cheezels	Snakes
Ben and Jerry’s	Boost Bar	M&M cookies	Nutella
Up and Go	Chocolate milk	Fruit cup cordial	Apple fruit drink
Bega string cheese	Cabanossi	Vegie sausages	White bread

There were 25 rating scales in total, composed of the two categories (Moral and Health), four demographic ratings and the distractor items. Responses for each scale were given on bipolar 7-point category scales. The Moral category was composed of eight ratings (anchors being – [scale 1] Tolerant of others/Intolerant of others, [scale 2] Sexually monogamous/Sexually promiscuous, [scale 3] Considerate/Inconsiderate, [scale 4] Virtuous/Immoral, [scale 5] Disciplined/Undisciplined, [scale 6] Pure/Polluted, [scale 7] Follows norms/Breaks norms, and [scale 8] Low aspirations/High aspirations). The Health category was composed of five ratings (anchors being – [scale 1] Health conscious/Not health-conscious, [scale 2] Active/Inactive, [scale 3] Thin/Fat, [scale 4] Healthy/Unhealthy, and [scale 5] Attractive/Unattractive). There were four demographic ratings (anchors being – [scale 1] Feminine/Masculine, [scale 2] Educated/Uneducated, [scale 3] Wealthy/Not-wealthy, and [scale 4] Old/Young). There were eight distractor items (anchors being – [scale 1] Prefers familiarity/Willingness to experiment, [scale 2] Does not plan ahead/Plans ahead, [scale 3] Practical/Idealistic, [scale 4] Likable/Unlikable, [scale 5] Intelligent/Unintelligent, [scale 6] Hard working/Lazy, [scale 7] Methodical/Spontaneous, and [scale 8] Quiet/Talkative).

#### Procedure

Survey data were collected online using Qualtrics. Participants provided their age, gender, and a diet descriptor. The first part of the survey adopted an open-ended format. Participants were told they would be shown six sets of food products and asked to consider the sort of person who would buy, prepare, and eat these types of food. In each case, after viewing a set, they were asked to type in their response. No details were provided as to the length or type of response they should provide, but a response of some kind was required to advance to the next part of the survey. For every participant, two of the food sets were always drawn from the Natural Food Set, two from the Unnatural Food Set, and two from the Mixed Food Set (see **Table 1**). The specific sets used were pre-determined based on which of four versions of the survey participants were randomly allocated to. The order of set presentation was randomized separately for each participant.

The second part of the survey used a rating scale format. Participants were again asked to consider the characteristics of individuals who buy, prepare, and eat the type of food presented. They were then informed they were going to be shown six sets of food items, for which there are several rating scales to complete. The same food categories were used as in part 1, the difference being that these sets had not been viewed before. After viewing the first set of foods, participants were presented with the 25 bipolar category scales, for which they were required to rate the extent to which each one characterized the sort of person who would consume these types of food. The scales were presented in a fixed random order. The survey took approximately 30 min to complete.

#### Analysis

Three *a priori* categories were used for coding – Moral, Health, and Demographics, with these being based around their respective rating scale attributes. Participants generated two responses for each type of Food Set. If either or both responses were relevant to one of the three coding categories, they received a score of 1 and a 0 if not (the valence of the theme was also coded if relevant). Note that one response may contain themes that are relevant to more than one category. To assess categorization reliability, 20% of these data were re-coded by an independent rater blind to the study aims. The Phi coefficient revealed significant agreement for all coding categories, Moral (ϕ = 0.71, *p* < 0.001), Health (ϕ = 0.61, *p* < 0.001), and Demographics (ϕ = 0.79, *p* < 0.001).

The Cochran’s *Q* test was used to compare the pattern of responses across food sets. A separate Cochran’s *Q* test was performed for each category – Moral, Health, and Demographics. Due to the omnibus nature of Cochran’s *Q* test, significant results were followed up with a planned comparison between the Natural and Unnatural Food Sets, using the McNemar Test.

Mean responses for the Natural, Unnatural, and Mixed Food Sets were computed by collapsing across all of the rating scales for the Moral and Health categories, respectively (with appropriate items reverse coded). Reliability (coefficient alpha) was calculated for these two categories, for each of the three food sets. Reliability was good for the Moral category (alphas 0.70-0.86); however, two items, Sexually monogamous/Sexually promiscuous and Follows norms/Break norms, had consistently low item total correlations (*r*’s, respectively, 0.18-0.38 and 0.02-0.47). We analyzed the Moral category data both with and without these two scales. As the outcomes were largely identical, and as it was our original intention to analyses these items together, we retained all eight scales. Reliability for the Health category (alphas 0.82-0.83) was good. For the Demographic category, each item was analyzed separately.

The two categories (Moral and Health), and the four demographic ratings, were each analyzed separately, using one-way repeated-measures ANOVAs with Food Set (Natural vs. Mixed vs. Unnatural) as the within-subject factor. Any main effect of Food Set, was followed up with a planned comparison between the Natural and Unnatural Food Sets. Huynh-Feldt corrections are reported for violations of sphericity.

The dataset is provided in the **Supplementary Material**.

### Results

#### Open-Ended Responses

Cochran’s *Q* test revealed a significant difference between mentions of moral themed responses between the Natural (20.2%), Unnatural (6.0%), and Mixed (6.0%) Food Sets, Q(2) = 13.71, *p* = 0.0011. The McNemar Test, revealed a significant difference between the Natural and Unnatural Food Sets, *X*^2^(1) = 7.56, *p* = 0.006. As predicted, there were more references to moral related person characteristics in the Natural Food Set. Moreover, within the Natural Food Set, 87.1% of mentions of moral person characteristics were positive. All references to moral person characteristics in the Unnatural Food Set were negative.

Across categories, 66.6% of participants mentioned health related themes when characterizing consumers of the Natural Food Set, 60.7% for the Unnatural Food Set, and 42.9% for the Mixed Food Set. Mentions of health significantly differed across the three categories, *Q*(2) = 25.00, *p* < 0.001. While there was no significant difference between the Natural and Unnatural Food Sets, there was a clear difference in valence. 97.1% of the judgments for the Natural Food Set were positive (i.e., healthy), while 83.1% of judgments for the Unnatural Food Set were negative (i.e., unhealthy).

There was no difference in mentions of demographic attributes across Sets.

#### Rating Scale Responses

For the Moral category, there was a main effect of Food Set, *F*(1.66, 137.64) = 104.73, *p* < 0.001, ${\text{η}}_{\text{p}}^{2}$ = 0.56. Contrasting responses on the Natural (*M* = 4.9, *SD* = 0.7) and Unnatural (*M* = 3.6, *SD* = 0.5) Food Sets revealed a significant difference, *t*(83) = 11.67, *p* < 0.001, indicating that participants felt that consumers of natural foods were more virtuous.

For the Health category, there was a main effect of Food Set, *F*(1.77, 147.24) = 326.15, *p* < 0.001, ${\text{η}}_{\text{p}}^{2}$ = 0.80. The planned contrast revealed a significant difference, *t*(83) = 21.22, *p* < 0.001, with consumers of Natural Food judged as healthier (*M* = 5.6, *SD* = 0.7), than consumers of Unnatural Food (*M* = 2.8, *SD* = 0.7).

Each of the demographic variables was analyzed separately. For gender, there was a main effect of Food Set, *F*(1.75, 145.47) = 52.91, *p* < 0.001, ${\text{η}}_{\text{p}}^{2}$ = 0.39. The planned contrast revealed that Natural Food consumers were judged to be more feminine (*M* = 3.2, *SD* = 0.9), than Unnatural Food consumers (*M* = 4.5, *SD* = 0.8), *t*(83) = 8.51, *p* < 0.001. For wealth, there was a main effect of Food Set, *F*(2, 166) = 110.22, *p* < 0.001, ${\text{η}}_{\text{p}}^{2}$ = 0.57. The planned contrast revealed that Natural Food consumers were judged to be wealthier (*M* = 5.1, *SD* = 0.9), than Unnatural Food consumers (*M* = 3.3, *SD* = 0.9), *t*(83) = 13.20, *p* < 0.001. For education, there was a main effect of Food Set, *F*(1.68, 139.37) = 100.29, *p* < 0.001, ${\text{η}}_{\text{p}}^{2}$ = 0.55. The planned contrast revealed that Natural Food consumers were judged to be better educated (*M* = 5.5, *SD* = 1.0) than Unnatural Food consumers (*M* = 4.1, *SD* = 0.8), *t*(83) = 10.44, *p* < 0.001. Finally, for age, there was a main effect of Food Set, *F*(2, 166) = 20.38, *p* < 0.001, ${\text{η}}_{\text{p}}^{2}$ = 0.20. The planned contrast revealed that Natural Food consumers were judged to be older (*M* = 3.6, *SD* = 1.2), than Unnatural Food consumers (*M* = 2.6, *SD* = 1.1), *t*(83) = 5.20, *p* < 0.001.

### Discussion

Study 1 sought to identify the person characteristics associated with consumers of natural foods, relative to unnatural foods and mixed food sets. The results from both the open and closed formats indicated that consumers of natural foods were judged to be more virtuous and healthier than consumers of unnatural food. In addition, the rating scale data revealed that natural food consumers were also regarded as more feminine, educated, wealthy, and older. To the extent that natural foods are health foods, these findings echo those of Stein and Nemeroff (1995), who found that knowing that someone consumes health foods elicits multiple positive impressions, including being more virtuous. However, an important and untested question remains. If participants really hold these beliefs about consumers of natural foods, especially that they are virtuous, then they should presumably trust them more than someone who consumes unnatural foods. Study 2 set out to explore this question.

## Study 2

Study 2 used an experimental design to determine if knowing about a target’s natural/unnatural food intake would influence trust-related behavior toward that target person. Participants were invited to play multiple rounds of the Trust Game (Hillebrandt et al., 2011). The Trust Game involves the exchange of tokens between two players. One player – the sender – starts the game with 10 tokens (later redeemable for cash) and has to decide how many tokens to send to the other player – the receiver. Importantly, players are informed of the following rule. The number of tokens the sender remits to the receiver *will be tripled* with both parties knowing that the other is aware of this rule. Once receiving the tripled amount, the receiver must decide whether to keep it all or cooperate and remit back the same or additional tokens to the sender. Therefore, the more the sender trusts the receiver, the more tokens they should send over the course of the game.

In our experiment, participants were provided with incidental information about the receiver, *via* some of their belongings left in the testing room. These belongings included some shopping, which either contained a set of natural or unnatural foods. In reality, the receiver was fictional and participants did not formally learn about this until the end of the experiment, when they were progressively debriefed and re-consented. An important consideration here was the number of tokens that should be returned by the fictitious receiver (i.e., by the experimenter) on each trial. We chose to follow a neutral rule, sending back as many tokens as the sender sent.

Operating under the premise that people who consume natural foods are perceived as more virtuous, we predicted that individuals playing against a natural food consuming receiver would send more tokens over the course of the game. By contrast, senders should be wary of unnatural food consuming receivers, if they believe that they are less virtuous. These presumed moral characteristics were assessed in the final phase of the experiment. First, participants were asked to judge what they thought the receiver was like (using the same 25 rating scales as per Study 1). Second, these same evaluations were completed again after participants’ attention was drawn to the fictional receiver’s shopping.

### Methods

#### Participants

Participants were Macquarie University undergraduates, who took part for course credit. As we had found, in Study 1, a medium to large effect size for spontaneous mentions of moral themes (*d* = 0.65) and a very substantial effect size for ratings of moral themes (*d* = 2.12), we estimated that the effect size for Study 2 would be large. With *d* set at 0.9 and with power at 80%, this would require a sample size of 20 per group. Consequently, we tested 40 participants (25 women) aged between 18 and 34 years (*M* = 20.2, *SD* = 3.7).

This study was carried out in accordance with the recommendations of the Macquarie University Human Research Ethics Committee (MQ HREC). The protocol was approved by the MQ HREC. All subjects gave written informed consent in accordance with the Declaration of Helsinki. Initial written consent was obtained to play the Trust Game and to make ratings about the other player, but without any mention of food, naturalness, the fictional status of the other player or the actual rationale for the study. At the end of the experiment, participants were progressively debriefed and then re-consented (noting that all who participated re-consented).

#### Materials

**FIGURE 1:**
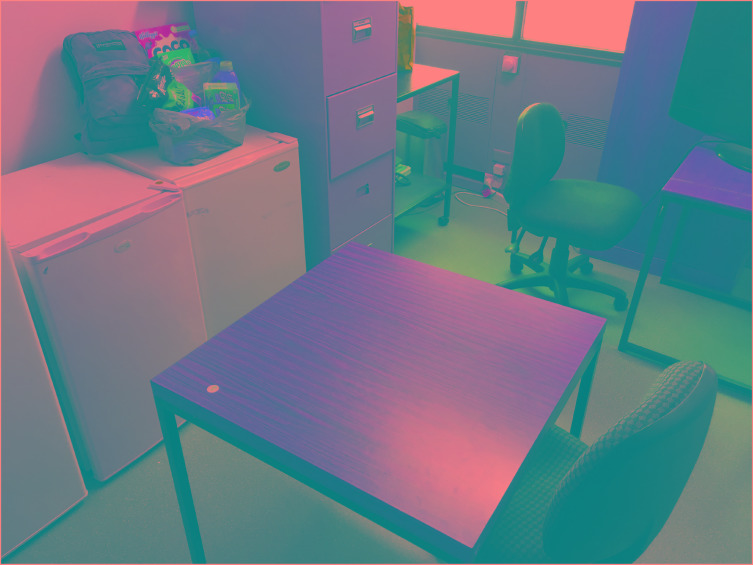
The fictitious receiver’s belongings — bag and shopping — can be seen on the top of right-hand side bar fridge. Participants were asked to place their belongings next to this on the adjacent bar fridge and were seated in the chair and desk displayed in the bottom mid-right foreground of the figure.

The food items used in Study 2, all vegetarian to minimize any differences on this variable, were drawn from the lists of natural and unnatural foods identified in the pilot. The natural foods were: green tea, coconut water, coconut oil, traditional oats, free range eggs, raw mixed nuts, almond spread, honey, dried apricots, canned beetroot, and a bag of limes. The unnatural foods were as follows: Cheezels, Kettle potato chips, Crispy chicken shapes, M&M cookies, Coco pops, Coca-cola, Fruit cup crush cordial, Up and go liquid breakfast, Mi goreng instant noodles, Margherita frozen pizza, and Bega string cheese. The respective foods were each placed in a shopping bag, with the contents readily visible from the participant’s chair (see **Figure 1**).

#### Procedure

The key manipulation was whether participants had a receiver who consumed natural or unnatural foods. This manipulation was instantiated by placing the shopping bags containing the natural or unnatural foods on top of a bar fridge in a testing room that contained basic furniture items (see **Figure 1**). This bar fridge was directly opposite where the participant sat, and was at eye height; thus, it was in their direct line of sight. When the participant arrived, they were asked to place their belongings on top of an adjacent bar fridge and next to the fictional receiver’s belongings – this being mentioned in passing by the experimenter during their welcome to the study. Whether the participant encountered a natural or unnatural food consuming receiver was determined by a fixed randomization schedule.

Participants were presented with written instructions about the Trust Game, followed by verbal reiteration/quizzing to ensure understanding. All participants were informed they had been randomly allocated to the role of sender. They were also told that the accumulated tokens earned across the four rounds of the Trust Game would be converted into real money at the end of the study. Participants were then left for a couple of minutes, while the experimenter purportedly explained the rules to the receiver. Upon returning, the experimenter then initiated the first round of the Trust Game.

The participant (i.e., the sender) was handed 10 tokens by the experimenter and given a minute to decide how many they wished to send to the receiver. They were asked to place the tokens in an envelope, which was then handed to the experimenter. Immediately following this transaction, the sender was asked to indicate how many tokens they expected to be returned by writing down the expected number. They were then asked to evaluate how confident they were about their judgment on a 5-point scale (anchors “Extremely Unconfident” to “Extremely Confident”). The experimenter then excused themselves to take the tokens to the fictional receiver and entered the adjacent laboratory where the receiver was supposedly located. The experimenter recorded the number of tokens sent and then placed the same number of tokens in a new envelope and returned this to the participant (i.e., the sender) in the other room. The participant (i.e., the sender) was then asked to count the number of tokens returned and place them in a glass jar. The experimenter then indicated this was the end of the first round. All steps of this process, except the instructions, were then repeated for a further three rounds. At the end of the final round, participants were told that the conversion rate would be $1 for 20 tokens, which would be received after the completion of a few questionnaires.

Participants were then asked to record their age, gender, and diet type. After this, they were asked to rate the receiver using same the list of 25 person characteristics described for Study 1 (in the same fixed random order) but completed using a paper and pencil format on 5-point scales (anchors as per Study 1). Immediately after completion, participants were required to fill out a second evaluation sheet. This evaluation drew the participants attention to the receiver’s belongings – namely their food shopping – and then asked the participant to consider what sort of person would “buy, prepare, and eat” these types of food using the same set of scales. Finally, participants were progressively debriefed by asking them whether they thought the receiver was real, and if they had doubts, when and why these doubts had arisen, and at the end, what they thought the purpose of the experiment was. The experiment took approximately 30 min to complete.

#### Analysis

The main dependent variable was the number of tokens sent by the sender on each of the four rounds of the game. These data were analyzed using a two-way mixed ANOVA with Round as the within-participant factor and Receiver Food Group (Natural vs. Unnatural) as the between factor. A further ANOVA was then performed including a new between factor, namely, whether participants reported during debriefing being aware or suspicious (or not aware or suspicious) as to the reality of the receiver. Huhn-Feldt corrections are reported where violations of sphericity occurred.

The expected token return data were significantly skewed and kurtotic and not amenable to transformation. As such, a Mann-Whitney test was used to compare the expected returns of each Receiver Food Group (Natural vs. Unnatural) at each level of Round (Rounds 1-4). Non-parametric trend tests (Page’s) were then run, to examine if there were changes across rounds in expected token returns, within each Food Group.

Confidence ratings were coded -2 “Extremely Unconfident” to 2 “Extremely Confident.” Initial analysis revealed these data to be significantly skewed and kurtotic, and not amenable to transformation. The same non-parametric approach, as above, was adopted.

Participants were asked to judge the characteristics of the receiver (Questionnaire 1) and then the type of person who would consume the foods identified as belonging to the receiver (Questionnaire 2). The 25 attribute ratings were dealt with in the same manner as for Study 1. The Moral category had adequate to good reliability (coefficient alphas of 0.68 [Questionnaire 1] and 0.80 [Questionnaire 2]). As with Study 1, the same two items (Sexually monogamous/Sexually promiscuous and Follows norms/Break norms), again had low item total correlations, and so we analyzed the Moral category data both with and without these two scales. As the outcomes were again largely identical, we retained all eight scales, as with Study 1. The Health category also had adequate to good reliability (coefficient alphas of 0.57 [Questionnaire 1] and 0.73 [Questionnaire 2]).

Independent samples *t*-tests were run to compare the Moral and Health categories, as well as the demographic ratings, by Food Group, for both Questionnaires 1 and 2. Finally, we examined whether these evaluations of receiver characteristics were associated with participants token sending behavior (as measured by the slope coefficient from fitting a straight line to each participants token sending across rounds) using stepwise regression.

The dataset is provided in the **Supplementary Material**.

### Results

#### Trust Game

Analysis of the tokens sent revealed a significant main effect of Receiver Food Group, *F*(1,38) = 4.69, *p* = 0.037, ${\text{η}}_{\text{p}}^{2}$ = 0.11, but no main effect of Round. This was qualified by an interaction between Receiver Food Group and Round, *F*(2.3,87.2) = 4.06, *p* = 0.016, ${\text{η}}_{\text{p}}^{2}$ = 0.10. As can be seen in **Table 2**, the natural Receiver Food Group tended to send the same number of tokens as rounds progressed with no significant change across rounds (test of linear trend, *p* = 0.30). By contrast, the unnatural Receiver Food Group progressively sent fewer tokens as rounds progressed (test of linear trend, *p* = 0.038).

**Table 2:** Mean (standard deviation) tokens sent, tokens expected to be returned, and confidence in this judgment, for groups with fictional receivers who either consume natural or unnatural foods.

Group			
Round	Tokens sent	Expected returns	Confidence
**Natural food group**			
Round 1	5.5 (2.3)	6.1 (4.7)	0.2 (0.7)
Round 2	6.0 (2.2)	6.9 (3.8)	0.4 (0.7)
Round 3	5.4 (3.2)	5.3 (3.4)	1.1 (0.7)
Round 4	6.8 (3.4)	5.8 (3.7)	1.1 (1.0)
**Unnatural food group**			
Round 1	5.2 (2.4)	6.5 (4.7)	0.3 (0.7)
Round 2	6.0 (2.7)	5.9 (4.1)	0.5 (0.5)
Round 3	4.4 (2.8)	4.5 (2.4)	1.1 (0.6)
Round 4	3.3 (3.0)	3.6 (4.5)	1.3 (0.9)

No participant identified the purpose of the study, but 17/40 participants (42.5%) – 8 out of 20 in the natural Receiver Food Group (40%) and 9 out of 20 (45%) in the unnatural Receiver Food Group – reported that they believed or suspected the receiver might be fictitious. We repeated the above ANOVA, now including Awareness as a factor. This revealed no effects involving Awareness, and the same main effect and interaction noted above, were again observed.

A Mann-Whitney test revealed no significant difference in the number of tokens expected to be returned (see **Table 2**) between the natural and unnatural Food Groups across Round 1 (*U* = 191.00, *p* = 0.806), Round 2 (*U* = 161.50, *p* = 0.293), and Round 3 (*U* = 168.00, *p* = 0.382). However, the expected token return for Round 4 significantly differed, *U* = 128.00, *p* = 0.050. Specifically, the number of tokens expected to be returned during Round 4 was greater for the natural Receiver Food Group than for the unnatural Receiver Food Group. Page’s test was run to examine whether the expected number of tokens returned changed across rounds within each group. For the natural Receiver Food Group, there was no significant change in expected returns across Rounds (*Z* < 1). For the unnatural Receiver Food Group, there was a significant drop in expected returns across the four rounds, *Z* = 3.06, *p* < 0.001.

Mann-Whitney tests revealed no significant difference in participant’s confidence ratings between Receiver Food Groups, on any rounds of the game (see **Table 2**). Page’s test was run to examine whether confidence ratings change across rounds within each Receiver Food Group. There was a significant increase in confidence ratings across rounds for both the natural Receiver Food Group (*Z* = 3.76, *p* < 0.001) and the unnatural Receiver Food Group (*Z* = 4.22, *p* < 0.001).

#### Characteristics of the Receiver

On Questionnaire 1 – judging what the receiver was like – *t*-tests revealed significant differences between the natural and unnatural Receiver Food Groups on the Health category, but not on the Moral category. For demographics, only education differed. Subjects in the natural Receiver Food Group judged their receiver more positively on health characteristics, and as being more educated, than participants in the unnatural Receiver Food Group (see **Table 3**).

**Table 3:** Characteristics of the fictional receiver (mean and SD) in the Trust Game as judged by participants.

Questionnaire			
Variable	Natural food group	Unnatural food group	*t*(38)=
**Questionnaire One (Fictional receiver characteristics)**			
Moral	3.6 (0.5)	3.4 (0.5)	1.06
Health	3.4 (0.5)	3.0 (0.3)	3.04^∗^
Gender	3.1 (0.9)	3.2 (0.8)	0.19
Wealth	3.4 (0.9)	3.2 (0.9)	0.87
Education	4.2 (0.8)	3.7 (0.6)	2.23^∗^
Age	2.6 (0.9)	2.3 (0.6)	1.44
**Questionnaire Two (Characteristics after pointing out food)**			
Moral	3.7 (0.4)	3.0 (0.6)	4.93^∗^
Health	4.1 (0.5)	2.4 (0.5)	10.43^∗^
Gender	2.4 (0.9)	3.5 (0.9)	3.95^∗^
Wealth	3.8 (0.9)	2.7 (0.7)	4.54^∗^
Education	4.2 (0.7)	3.1 (0.9)	4.47^∗^
Age	2.4 (1.0)	1.7 (0.7)	2.36^∗^

*^∗^p < 0.05.*

On Questionnaire 2 – being directed to take into account the receiver’s shopping – significant differences were now evident for all categories and demographic items (see **Table 3**). Participants in the natural Receiver Food Group rated their receiver more positively on the Moral and Health categories, and as more feminine, wealthy, educated, and older, relative to the unnatural Receiver Food Group.

#### Relationship Between Perceived Receiver Characteristics and Behavior on the Trust Game

Participants’ token sending behavior as measured by the linear slope coefficient across rounds was used as the dependent variable in a step-wise regression, with the two category scores and the four demographic variables from Questionnaire 1 (characteristics of the receiver) as predictors. The final model was significant *F*(1,39) = 6.72, *p* < 0.02, accounting for 12.8% of the variance in token sending behavior across rounds. Only one predictor was retained in this model – the moral rating category – this being positively associated with the slope coefficient (*r* = 0.39), such that higher scores across moral ratings about the receiver were associated with more positive slope coefficients (i.e., a tendency to send more tokens as rounds progressed).

We then repeated this analysis, now using the four category ratings obtained from Questionnaire 2 (i.e., taking the shopping into account). The outcome was the same. The final model was significant *F*(1,39) = 8.43, *p* < 0.01, accounting for 16.0% of the variance in token sending behavior across rounds. The only predictor retained in the model was the moral category; this again being positively associated with the slope coefficient (*r* = 0.43).

### Discussion

Study 2 determined whether people’s trust-related behavior was influenced by their impressions of another person, based on their likely consumption of natural or unnatural food. Participants in the natural Receiver Food Group persisted in sending tokens at the same rate across rounds presumably in the hope of a more favorable return, while those in the unnatural Receiver Food Group progressively sent fewer tokens as rounds progressed. This pattern of token sender behavior was related to the characteristics of the receiver *as perceived by the sender*. When asked to judge the receiver, only health and education differed between Receiver Food Groups. However, the moral rating category was the only significant predictor of token sending behavior across rounds. The same finding was obtained when participants were asked what sort of person would eat the food purportedly belonging to their receiver – again moral ratings were the only predictor of token sending behavior across rounds.

## General Discussion

We demonstrated for the first time in Study 1 that natural food consumers are indeed judged as more virtuous than unnatural food consumers, irrespective of whether this is measured using an open or closed format approach. Study 2 extended these findings using a novel behavioral approach, testing if the type of moral-related beliefs identified in Study 1 translated into behavior. Using the Trust Game (Hillebrandt et al., 2011), participants whose fictitious partner (termed the receiver) in the game consumed natural foods seemed to be trusted more, than participants whose fictitious receiver consumed unnatural foods. This was expressed through the number of tokens sent across the four rounds of the game (sending a consistent amount) and by the pattern of expected returns (expecting a consistent return). Finally, participants were asked to evaluate the characteristics of their fictitious receiver on the Trust Game. The moral ratings category was the only predictor retained in the step-wise regression model of token sending behavior across rounds.

Both studies indicate that participants view consumers of natural food as more virtuous than consumers of unnatural foods. We suggest there are three possible classes of explanation for this. The first is that participants beliefs and behavior about the superior moral attributes in natural food consumers derives from a more fundamental association between health or demographic attributes and natural food consumption. As we noted in the Introduction, healthy behaviors in general have positive moral connotations (e.g., Conrad, 1994; Hoverd and Sibley, 2007). There is also evidence that at least some demographic attributes do as well. Women are generally perceived as more empathetic and trustworthy than men (e.g., Chaudhuri, 2012), and they are perceived to exceed men on at least some moral dimensions (e.g., care orientation; Jaffee and Hyde, 2000). Older adults appear more trustworthy than younger adults (e.g., Castle et al., 2012). The point here is that if natural food consumers are primarily perceived as healthier, older, and female (or on any other similarly related dimension), it may be these differences that then drive more favorable moral judgments/behavior.

A second possibility, which is not exclusive to the first, is that participants base their judgments on their explicit knowledge about natural (or unnatural foods) irrespective of its correctness (for similar argument see Li and Chapman, 2012). If a participant knows that certain foods are less eco-friendly, and/or that some foods are not fair trade and/or that they may involve some form of animal or human exploitation, they may be more inclined to believe that someone who uses these products also knows this but chooses to ignore it or even condones it. This would seem to be a moral choice, and if a person can ignore one form of immorality, then presumably they may tolerate or ignore others. This idea garners support from research described in Section “Introduction,” in which non-vegetarian participants were seemingly aware of the moral motivations of vegetarians (Ruby and Heine, 2011) and that the environmental and altruistic connotations of natural foods account for some of their popularity (Honkanen et al., 2006). On this basis, participants’ moral judgments flow from their explicit knowledge of the potential goods and harms associated with different types of food and they infer that the consumer knows this. It is then but a short step to make additional inferences about their moral attributes.

There is a third and more esoteric possibility. The anthropologist James Frazer described three laws of sympathetic magic, two of which are pertinent here (Nemeroff and Rozin, 1989). One is the law of similarity, such that similar things have similar properties and the other is the law of contagion, such that once something has been in contact with something else, it remains in contact. Nemeroff and Rozin (1989) demonstrated how these two laws of sympathetic magic could apply to food, with participants asked to read about two tribes that differed only in what they ate (wild boars vs. turtles). When participants were asked to give their impressions of what these peoples would be like, the dietary information influenced their responding (e.g., the turtle eating tribe being better swimmers, and boar hunters being very strong). These findings were interpreted as being consistent with the notion that “you are what you eat” (i.e., eating wild boar makes you *boar*-like). By the same token, participants in our two studies may believe that consuming natural food (or in contrast unnatural food) imbues its eater with the same properties that are inherent in the food itself.

The two studies reported here did not set out to test between these alternatives; however, some of the findings certainly speak to the first and second explanations. If other correlated health or demographic factors were driving moral responses to naturalness, then the results from Study 2 would be problematic for this account. Here, the moral characteristics of the fictitious receiver were the best predictor of behavior on the Trust Game – not the health or demographic attributes. Needless to say, these characteristics were measured *after* the Trust Game (i.e., behavior on the Trust Game could have influenced these ratings), but this seems unlikely, as the fictitious receiver behaved in the same manner to all participants. In sum, these observations are not consistent with the idea that other correlated attributes of naturalness drive its positive moral overtones.

We would suggest that the second possibility above, one based upon explicit knowledge, would seem the more likely explanatory candidate. While we did not assess participant knowledge about natural food, it seems more parsimonious to explain our findings in this way. Indeed, such an account makes the eminently testable prediction that participants’ knowledge about natural foods, food processing, industrialized agriculture, etc., should have a direct bearing on their moral evaluations of natural foods and those who consume them.

In conclusion, and irrespective of the mechanism, our findings suggest that people believe that consumers of natural foods are more virtuous than consumers of unnatural (i.e., processed) foods, and that they act in accordance with these beliefs.

## Author Contributions

ZT and RS jointly designed, analyzed, and wrote up this study. ZT undertook all testing and data collection.

## Conflict of Interest Statement

The authors declare that the research was conducted in the absence of any commercial or financial relationships that could be construed as a potential conflict of interest.
